# Binary Polyamide-Imide Fibrous Superelastic Aerogels for Fire-Retardant and High-Temperature Air Filtration

**DOI:** 10.3390/polym14224933

**Published:** 2022-11-15

**Authors:** Yuezhen Hua, Wang Cui, Zekai Ji, Xin Wang, Zheng Wu, Yong Liu, Yuyao Li

**Affiliations:** 1 School of Textile Science and Engineering, Tiangong University, Tianjin 300387, China; 2 Nantong Bolian Material Technology Co., Ltd., Nantong 226010, China

**Keywords:** polyamide-imide, structural construction, air filtration, binary aerogels

## Abstract

Fibrous air filtration materials are highly desirable for particle removal from high-temperature emission sources. However, the existing commercial filter materials suffer from either low filtration efficiency or high pressure drop, due to the difficulty in achieving small fiber diameter and high porosity simultaneously. Herein, we report a facile strategy to fabricate mechanical robust fibrous aerogels by using dual-scale sized PAI/BMI filaments and fibers, which are derived from wet spinning and electrospinning technologies, respectively. The creativity of this design is that PAI/BMI filaments can serve as the enhancing skeleton and PAI/BMI fibers can assemble into high-porosity interconnected networks, enabling the improvement of both mechanical property and air filtration performance. The resultant dual-scale sized PAI/PBMI fibrous aerogels show a compressive stress of 8.36 MPa, a high filtration efficiency of 90.78% (particle diameter of 2.5 μm); for particle diameter over 5 μm, they have 99.99% ultra-high filtration efficiency, a low pressure drop of 20 Pa, and high QF of 0.12 Pa^−1^, as well as thermostable and fire-retardant properties (thermal decomposition temperature up to 342.7 °C). The successive fabrication of this material is of great significance for the govern of industrial dust.

## 1. Introduction

The 2019 coronavirus disease (COVID-19) still constitutes the forefront of public health concerns based on the fact that more than 600 million confirmed cases were reported and there are almost 40,000 new cases a day [1,2]. Particularly, environmental exposure to fine particles with a diameter of less than 2.5 μm (PM_2.5_) tends to increase the risk of COVID-19 attack because of the particles’ ability to carry viruses and float on air [3,4]. To ensure the low level of PM_2.5_ in the air, it is important to remove particles from emission sources, involving power generation, coal combustion, industrial and agricultural emissions, and so on [5,6]. The common high temperature (50–250 °C) feature of emission sources lead to the high demand of air filtration materials with thermostable and fire-retardant properties [7,8].

Currently, the commercial high-temperature air filtration materials mainly include glass fibers, polyimide (PI) fibers, and aramid fibers, which all feature with micro-sized fiber diameters [9,10,11]. Resulting from the accumulation of fibers, the pores are relatively large, and lead to difficulties in achieving high filtration efficiency towards small but poisonous PM_2.5_ [12,13]. As an alternative, electrospun nanofibers have gained the attention of researchers, owing to their small fiber diameters, which would contribute to small pores and large specific surface area, all being beneficial to high air-filtration performance [14,15]. However, it should not be ignored that electrospun fibrous assemblies tend to show a dense packing architecture, which would give rise to challenges in decreasing pressure drop and increasing the dust loading capacity. Therefore, fabricating thermostable electrospun nanofibrous assemblies with high porosity is highly recommended.

Fibrous aerogels have been acknowledged due to their ultrahigh porosity, which generally can easily achieve 99%. Furthermore, assembling electrospun nanofibers to create aerogels would enable the formation of interconnected channels, scalable pores, and large specific surface areas [16,17]. All of the above characteristics are beneficial for maintaining a relative balance between high filtration efficiency and low pressure drop, and also can contribute to a high dust-holding capacity [18,19]. Although PI fibrous filters are the leading product in the air filtration market, there are almost no PI nanofibrous aerogels reported, which might be because the intrinsic insoluble property of PI makes electrospun nanofibers hard to acquire [20,21]. Our previous work proved polyamide-imide (PAI) can be the alternative material of PI, owing to its similar molecular structure and thermostable property, and, more importantly, its good solubility. However, the already-prepared PAI nanofibrous aerogels have to contain stiff 15 wt% SiO_2_ fibers in order to meet the requirement of satisfying mechanical property. The tedious procedures and harsh conditions for preparing stiff SiO_2_ fibers limit their practical application in high-temperature air filtration [22].

Herein, we constructed dual-scale sized PAI/PBMI fibrous aerogels based on the fabrication of PAI/BMI filaments and fibers, which were obtained from wet spinning and electrospinning processes, respectively. PAI/BMI content in the wet spinning solution was regulated first to obtain PAI/BMI filaments with a large diameter and robust mechanical properties. PAI/BMI filaments were further combined with electrospun PAI/BMI fibers with small diameters and experienced dispersion, freeze-drying, and crosslinking procedures, resulting in dual-scale sized PAI/PBMI fibrous aerogels. PAI/BMI filaments, as the enhancing skeleton, were introduced into the electrospun PAI/BMI fiber networks with an optimized weight ratio. The resultant dual-scale sized PAI/BMI fibrous aerogels showed competitive comprehensive performances including high filtration efficiency, low air resistance, thermostability, and fire-retardance.

## 2. Materials and Methods

### 2.1. Materials

Polyamide-imide (PAI) with a molecular weight of 20,000 was bought from Nantong Bolian Material Technology Co., Ltd. (Nantong, China), and another PAI with the molecular weight of 37,000 was purchased from Solvay S.A., USA; N,N-1,4-bismaleimide (BMI) and tertiary butanol were provided by Aladdin Chemistry Co., Ltd., Shanghai, China; N,N-dimethylformamide (DMF) was purchased from Tianjin Kailis Fine Chemical Co., Ltd., Tianjin, China. All chemicals were of analytical grade (AR) and used directly without further purification.

### 2.2. Preparation of Spinning Solution

The preparation of the spinning solution in this experiment involves two systems:

(1) Feasibility analysis of wet spinning. The specific operation steps are as follows: the PAI (Solvay, Mw = 37,000) and BMI powders with different mass ratios were added into DMF, and stirred for 0.5 h at room temperature to obtain a clear and transparent uniform gelatinous liquid. The prepared different spinning solutions are shown in Table S1 (Supplementary Materials).

(2) Electrospun solution preparation. The specific operation steps are as follows: weigh a certain quality of PAI and BMI powder and add them to DMF, stirring them for 10 h at room temperature to obtain a clear and transparent spinning precursor solution. The content of PAI (Bolian, Mw = 20,000) and BMI in the electrospinning solution were 34 wt% and 6.8 wt%, respectively.

Among them, BMI was used as a small molecule cross-linking agent in the later process to conduct thermal cross-linking with PAI to build PAI/BMI semi-interpenetrating polymer network (semi-IPN).

### 2.3. Preparation of Wet-Spun PAI/BMI Filaments

The spinning solution was pumped into a solidification tank with tap water at a rate of 2 mL h^−1^ using a 23 G needle, and then directly wound on a receiving roller operating at a constant speed of 5 rpm after solidification. After spinning, the filament was placed in a vacuum oven, heated to 60 °C, and kept at a constant temperature for 2 h to remove residual solvent. After natural cooling, the PAI/BMI filament was obtained.

### 2.4. Preparation of Electrospun PAI/BMI Fibers

The prepared spinning solution was loaded into three syringes capped with 20 G needles, and the pumping-out rate of solutions was fixed at 1 mL h^−1^. A high and constant voltage of 20 kV was applied to trigger the transformation of solution to continuous jets. The charged jets experienced a flight distance of 20 cm, which is ensured by moving the needle tips far away from or near the collector. In addition, the sliding table carrying the rotating solution moved horizontally at a speed of 30 cm min^−1^, and the speed of the collector was 50 rpm. PAI/BMI fibers were deposited on stainless steel ground-receiving rollers. During the electrospinning process, the ambient temperature was controlled at 25 ± 3 °C and the relative humidity was controlled at 30 ± 5%.

### 2.5. Preparation of Dual-Scale Filament/Fiber Aerogels

The fabrication of dual-scale sized PAI/BMI fibrous aerogel mainly involves three steps: homogeneous dispersion, freeze-drying, and bond cross-linking. The electrospun fiber membrane was cut into small pieces with side lengths of about 5 mm, and the wet-spun filaments were cut into short fibers of 5 mm, and these were immersed in 100 mL of water/tert-butanol mixture with a weight ratio of 1/4. The mixture was homogenized and dispersed at 2800 rpm for 0.3 min in a beater to obtain uniform fiber dispersion. The mixture was then transferred to a freezing mold in a liquid nitrogen environment at −196 °C to achieve rapid freezing of the dispersion. The uncross-linked fiber aerogels were obtained after the ice crystals were withdrawn from the frozen body by a vacuum dryer, which was further placed at 200 °C for 2 h to induce the in-suit polymerization of BMI. Thus, the dual-scale PAI/PBMI fibrous aerogels were obtained.

### 2.6. Characterization

The microstructure of wet-spun filaments, electrospun fiber, and dual-scale PAI/PBMI aerogels was observed by optical microscopy (K-ALPHA, Thermo Fisher Scientific, Waltham, MA, USA) and scanning electron microscopy (Phenom XL, 10 keV, Phenom-World, Eindhoven, The Netherlands). The thermal stability of the material was characterized by a comprehensive thermal analyzer (STA 449F5(TG-DSC). YG005E electronic single-yarn strength tester was used to test the tensile properties of wet-spun filament, test details are provided in the Section S1 (Supplementary Materials). The compression properties of aerogels were characterized by Keithley 2400 Flexible Material Tester, as described in Section S2 (Supplementary Materials). The filtration performance was measured using a filter tester (LZC-K, BDA Filtration Technology Co., Ltd., Suzhou, China) as described in Section S3 (Supplementary Materials).

## 3. Result and Discussion

### 3.1. Fabrication of Wet-Spun PAI/BMI Filaments

**Figure 1:**
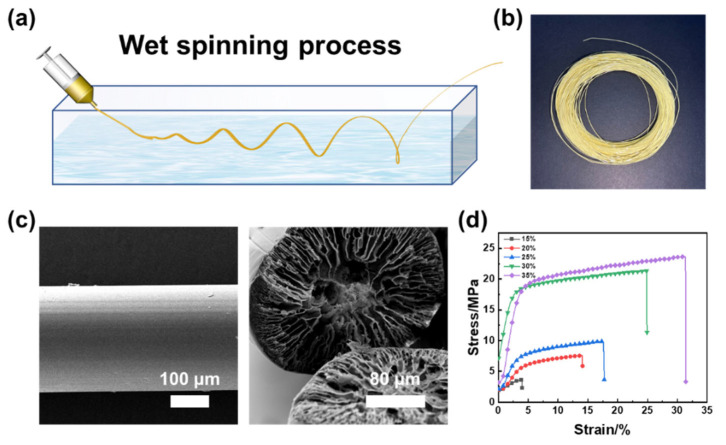
(**a**) The process of fabrication of PAI/BMI filaments. (**b**) PAI filaments. (**c**) The SEM images of PAI/BMI filament. (**d**) Stress-strain curves of filaments with different PAI content.

In order to fabricate superelastic and durable aerogels, a new strategy is proposed to prepare PAI/BMI (Polyamide-imide/N,N-1,4-bismaleimide) fibrous aerogels with dual-scale fiber diameters. Firstly, high strength PAI filament was prepared by the wet spinning process as the reinforcement of fibrous aerogels. The fabrication process of wet-spun PAI filaments is shown in Figure 1a. During the wet spinning process, the bidirectional diffusion between the solvent (DMF) and coagulation bath (water) led to the phase separation between polymer and solvents, which resulted in the formation of filaments. At the same time, due to the slow diffusion between the DMF and water, the filaments’ forming process was relatively mild, resulting in the formation of the circular cross-section. In addition, when the spinning solution met with the coagulation bath, the filaments cortex was formed quickly, and some defects or cracks appeared on the surface of the filaments. The cortex contraction rate was small, while the core contraction rate was large, producing a flaw and a cavity between the cortex and the core [23,24]. After the primary filaments were stretched by the subsequent process, the micropores were elongated in the shape of a shuttle (Figure S1, Supplementary Materials). The obtained PAI filaments are shown in Figure 1b. The filaments presented with a yellow luster, smooth surface, circular cross-section, and porous structure inside.

Figure 1c shows the electron microscopy (SEM) images of filaments and the SEM images of different PAI contents are shown in Figure S2 (Supplementary Materials). The filaments’ diameter showed negligible changes, however, the internal hole of the filaments decreased with the increase of PAI content. The reason for this was that when the concentration of the solution increased, the diffusion behavior of each molecule became harder, and then the diffusion coefficient of the solvent and coagulation bath decreased. Thus, as the PAI content increased, the higher the total solid content of the filament, the less likely it was to generate holes.

The stress-strain curves of filaments with different PAI contents are shown in Figure 1d. The tensile strength of the PAI filament first increased slightly when the PAI contents were less than 30 wt%. When the PAI content was more than 30 wt%, the viscosity of the spinning solution was too high, and the spinning could not be rapidly and stably produced. On the whole, when the PAI solute content was 30 wt%, the filaments had a breaking strength of 23 MPa, and the filaments preparation process still maintained good spinnability, which has both performance and cost advantages.

In addition, we further improved the mechanical properties of filaments by doping BMI into spinning solution. Figure S3 showed the tensile modulus of PAI filaments with different BMI contents before and after heat curing. The increased BMI content was beneficial to the improvement of filament strength, and the mechanical properties of filaments were greatly improved after heat curing. The reason for this was that, on the one hand, the in situ self-polymerization of the BMI monomer occurred in the thermal environment, generating a semi-interpenetrating polymer network (semi-IPN) that interweaves PAI and BMI on a molecular scale [25]. On the other hand, with the increase of BMI content, the solid content per unit length of filament increased, which improved the mechanical properties of the filaments. When the BMI content was 9 wt%, the tensile modulus had been greatly improved, and could reach 78.04 MPa.

### 3.2. Preparation of Dual-Scale Filament/Fiber Aerogels

**Figure 2:**
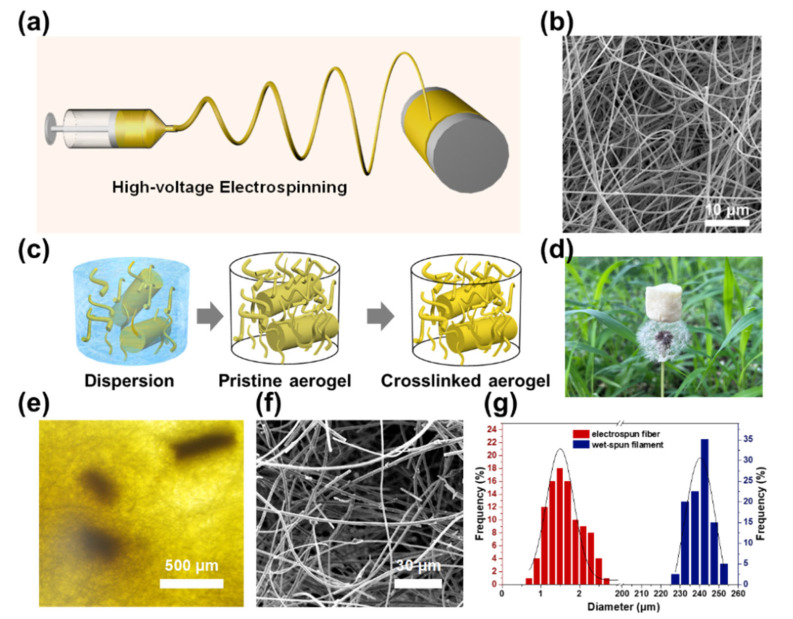
(**a**) The process of fabrication of PAI/BMI electrospun fiber. (**b**) The SEM images of PAI/BMI electrospun fibers. (**c**) The schematic diagram of PAI/PBMI aerogel preparation process. The PAI/PBMI aerogel (**d**) optical image and (**e**) SEM image. (**f**) Distribution of filaments and fibers within PAI/PBMI aerogel. (**g**) The diameter distribution of the PAI/PBMI aerogels.

PAI/BMI micro-sized fibers were prepared as the matrix of aerogels via electrospinning technology. The preparation process of the electrospun fibers is shown in Figure 2a. Under a high-voltage electric field, polymer solution pulled from the tip of the syringe would experience phase separation and an elongating process at the same time, depositing on the collectors and showing a disorderly arrangement morphology, as shown in Figure 2b.

Figure 2c depicts the synthetic route of the dual-scale PAI/BMI fibrous aerogels. The aerogels were prepared by blending wet-spun filaments and electrospun fibers. These two materials were first homogenized in water/tert-butanol mixture to form well-dispersed fiber dispersions, followed by freeze-drying to form an unjointed architecture pristine PAI/BMI aerogel. Driven by the moving solidification front, fibers in the dispersion were repelled and accumulated gradually in the unoccupied space of the growing cytosolic solvent crystals. This was controlled by complex and dynamic liquid fibers and fiber–fiber interactions [26]. In order to further promote the physical bonding between fibers, the uncross-linked PAI/BMI fibers after freeze-drying were heated at 220 °C for 2 h to form a cross-linked fiber network, resulting in the synthesized PAI/Polybismaleimide (PBMI) fibers with attractive compression recovery properties (Supplementary Video; Figure S4, Supplementary Material). The peak at 1608 cm^−1^ was caused by the tensile vibration of C=C, which was greatly reduced after in situ crosslinking, indicating that most of the C=C bonds had polymerized [27]. The intensity of the peaks at 748 cm^−1^ and 590 cm^−1^ was weakened, which can be attributed to out-of-plane C-H bending vibration and out-of-plane C-O bending vibration [28,29]. Notably, the wet-spun filaments in the PAI/PBMI fibrous aerogels acted as a rigid support, enhancing the structural stability of the aerogels.

The obtained aerogel showed a low bulk density of 0.011 g/cm^3^ and a high porosity of 99.2%, the white pom-poms of dandelion seeds showed negligible deformation, while supporting the ultralight aerogel (Figure 2d). Optical microscopy and SEM image of the microstructure of the obtained dual-scale PAI/PBMI aerogels showed that filaments and fibers with totally different diameters existed in the aerogel (Figure 2e, Figure S5, Supplementary Materials). The micro-fibers inside the aerogel were firmly welded to the wet-spun filament scaffold to form a double network structure. The SEM image of the ultrafine fibers is shown in Figure 2f. The electrospun staple fibers are uniformly dispersed in the aerogel prepared after homogenous dispersion. The diameter distribution of the PAI/PBMI aerogels was evaluated, as shown in Figure 2g. The diameter of the electrospun fiber was mainly distributed from 2 to 4 μm, and the wet spinning filaments diameter was mainly distributed in 200~300 μm, illustrating that aerogels are composed of dual-scale sized fibers.

### 3.3. Compression Properties of Ultralight Composite Fiber Aerogel

**Figure 3:**
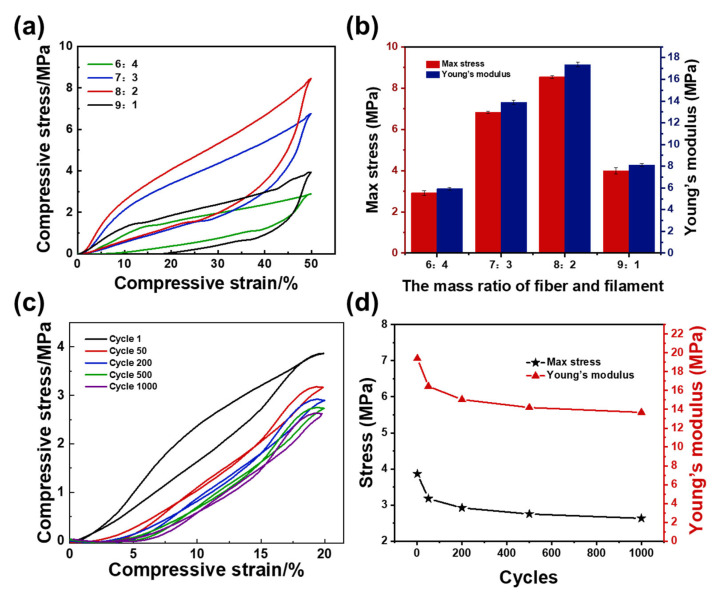
(**a**) The compression curves of different mass ratio of fibers/filaments aerogels. (**b**) The Young’s modulus, maximum stress of PAI/PBMI aerogels. (**c**) The cyclic compression mechanism and (**d**) Young’s modulus, maximum stress of PAI/PBMI aerogel.

The structural stability of filter media must be evaluated considering the continuous impaction from the airflow with high velocity. The compression property with a different mass ratio of fibers/filaments is shown in Figure 3a. All aerogels exhibited obvious nonlinear mechanical behavior, and there was no significant difference in the plastic deformation for the four aerogels after just one compression cycle. Interestingly, although the filaments were introduced as the enhancing skeleton, the maximum stress and the Young’s modulus of the aerogels were not found when the filament content reached the largest proportion of 40%, which could be because the stiff, short, and thick filaments cannot form an intertwined network. With the change of the mass ratio of fibers/filaments from 6:4 to 8:2, the maximum stress and the Young’s modulus increased to the highest value of 8.54 MPa and 17.36 MPa (Figure 3b), which might be attributed to the interaction network between the electrospun fibers combining more tightly. Afterwards, the compressive modulus of the aerogel was greatly reduced due to the reduction in the content of filaments used for the support structure in the aerogel, making it difficult to withstand large compressive stress. The above experimental results illustrated the contribution of “stiff-soft” fibrous networks on mechanical property.

The PAI/PBMI aerogel with mass ratio of fibers/filaments equal to 8:2 was subjected to 1000 loading and unloading fatigue cyclic compression tests with ε of 20% (which means the strain always maintains 20%) and a loading rate of 50 mm/min^−1^ (Figure 3c), which showed slight plastic deformation (3.34% for the 50th, 4.26% for the 1000th), and the structural robustness was outstanding. In contrast, typical polymer foams strained at 60% exhibited 20–30% plastic deformation, while other fibrous foams exhibited greater than 20% plastic deformation at similar strains [30,31,32]. Likewise, the stiffness or strength of PAI/PBMI aerogels did not decrease significantly after 1000 compression cycles, and retained more than 80% of the original Young’s modulus and maximum stress (Figure 3d). The dual-scale composite aerogel material had good compression recovery and could be used for a long time.

### 3.4. High-Temperature Air Filtration Application of Aerogels

**Figure 4:**
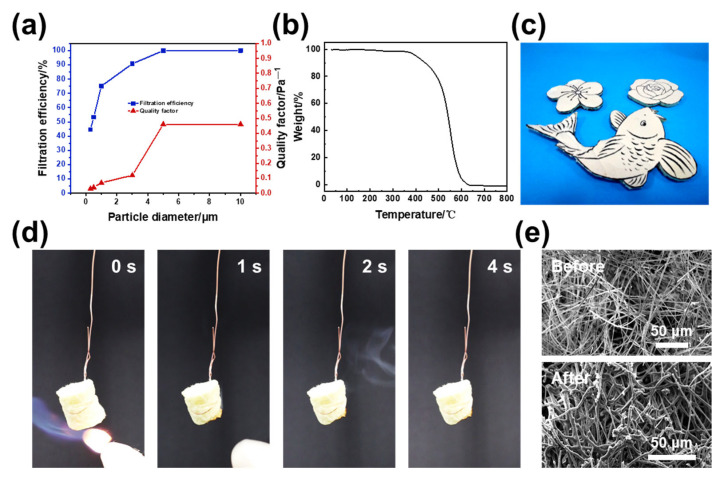
(**a**) Filtration efficiency. (**b**) TG curves. (**c**) An optical photograph of PAI/PBMI aerogel with diverse shapes. (**d**) Flame retardant performance demonstration. (**e**) The SEM image of PAI/PBMI aerogel before and after burning.

Considering the complex components of industrial dusts, we evaluated the air filtration of aerogels (the mass ratio of fibers/filaments equal to 8:2) towards particles with a wide range of sizes. Figure 4a shows the filtration efficiency of the PAI/PBMI aerogels for particles with particle sizes including 0.3, 0.5, 1, 3, 5, and 10 μm (PM_0.3_, PM_0.5_, PM_1_, PM_3_, PM_5_, and PM_10_). Obviously, higher filtration efficiencies could be reached while filtering larger particles, which illustrates the physical sieving mechanism of aerogels. In addition, for the PM_3_ accounting for the largest proportion within industrial dusts, aerogel materials exhibited a very high filtration efficiency of 90.78% (PM_2.5_) for fine particles while maintaining a low pressure drop of 20 Pa, and they showed a high quality-factor (QF) of 0.12 Pa^−1^, showing a cost-effective prospect in practical applications.

The thermostable property is very necessary for high-temperature air filters. Figure 4b shows the TG curve of the PAI/PBMI aerogel. The thermal decomposition of an aerogel is mainly divided into three stages. The first stage is 0~520 °C, during which the sample has only a slight weight loss, which is attributed to the loss of water molecules and other volatile small molecules in the sample. In the second stage (520~570 °C), significant weight loss occurs and the thermal degradation of aerogel components is serious. In the third stage, the residual carbon product continues to decompose at a high temperature to form the final residual carbon component. Because of the simplicity of the assembly process and the easy availability of electrospun fibers in our method, there is great versatility in controlling the shape of the PAI/PBMI aerogels. As shown in Figure 4c, PAI/PBMI integrated aerogels with desired shapes, such as plum blossom, peach blossom, and complex koi shapes, can be easily prepared. The demonstration process of the flame retardancy of aerogels is shown in Figure 4d. At the moment of contact with the high temperature flame, the aerogel burns slightly, but it goes out immediately after leaving the flame, and the fibers burn independently in the combustion process without dropping. According to the SEM images of aerogel materials before and after combustion (Figure 4e), most of the fibers in the aerogel materials remain independent as fibers after sintering at high temperature, while the conventional droplet polymer fibers show the form of molten film [33]. This is mainly because PAI polymer has excellent flame retardancy due to the existence of amide groups in the molecular chain.

## 4. Conclusions

In conclusion, we fabricated PAI/PBMI aerogels composed of dual-scale sized PAI/BMI filaments and fibers by exposing them to wet spinning and electrospinning process first. PAI/BMI filaments (with the average diameter of 239.83 μm) played the role of reinforcing skeletons, and PAI/BMI fibers (with an average diameter of 1.17 μm) served as the aerogel matrix. While the mass ratio of fibers/filaments was equal to 8:2, the maximum stress and Young’s modulus exhibited the highest values of 8.54 MPa and 17.36 MPa, respectively. In addition, the composite aerogels could bear 1000 compressive cycles with only 4.26% plastic deformation, implying their good structural stability. Dual-scale sized PAI/PBMI filaments/fiber aerogels also showed a high filtration efficiency of 90.78%. The resultant dual-scale sized PAI/PBMI fibrous aerogels showed a compressive stress of 8.36 MPa, a high filtration efficiency of 90.78% (particle diameter of 2.5 μm); particles with a diameter over 5 μm had 99.99% ultra-high filtration efficiency, a low pressure drop of 20 Pa, high QF of 0.12 Pa^−1^, and attractive thermostable and fire-retardant properties (thermal decomposition temperature up to 342.7 °C), showing great potential in the field of high temperature filtration.

## Data Availability

Not applicable.

## Supplementary Materials

The following supporting information can be downloaded at: https://www.mdpi.com/article/10.3390/polym14224933/s1, Figure S1: The SEM image of cross-section of PAI/BMI filament, Figure S2: SEM images of different PAI content, Figure S3: Tensile modulus of PAI/BMI filament before and after crosslinking, Figure S4: FTIR spectra of BMI powder before and after heating polymerization, Figure S5: The SEM image of dual-scale PAI/PBMI aerogel. Table S1: The prepared different spinning solutions of wet spinning filaments. Supplementary Video: The compression recovery property of dual-scale PAI/PBMI fibrous aerogels. Supplementary Methods: Section S1 single yarn strength tester, Section S2 compression properties test of aerogels, Section S3 filtration performance.
