# Supplementary material for: Binary Polyamide-Imide Fibrous Superelastic Aerogels for Fire-Retardant and High-Temperature Air Filtration
Source: Polymers (Basel). 2022 Nov 15;14(22):4933. doi: 10.3390/polym14224933 (PMC9692261; doi:10.3390/polym14224933)
Supplement: Supplementary file 1 [file polymers-14-04933-s001.zip › polymers-1952795-supplementary.pdf]

Supplementary Material for

Binary Polyamide-Imide Fibrous Superelastic Aerogels for Fire-Retardant and High-Temperature Air Filtration

Yuezhen Hua [1], Wang Cui [1], Zekai Ji [2], Xin Wang [1], Zheng Wu [1], Yong Liu [1] and Yuyao Li [1,*]

[1] School of Textile Science and Engineering, Tiangong University, Tianjin 300387, China
[2] Nantong Bolian Material Technology Co., Ltd., Nantong 226010, China
* Correspondence: liyuyao@tiangong.edu.cn

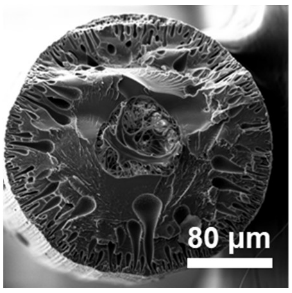

Figure S1. SEM images of cross-section of PAI/PBMI filament.

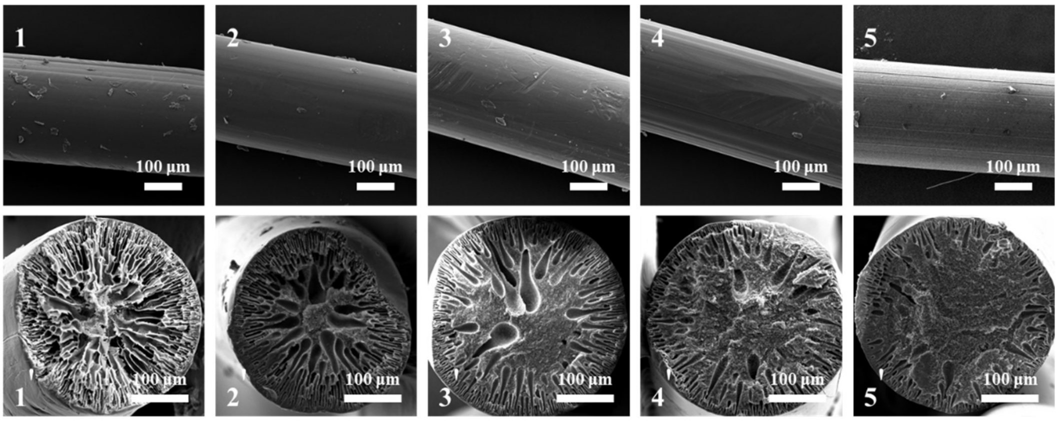

Figure S2.SEM images of different PAI content.(From left to right, the content of PAI is 15-35%)

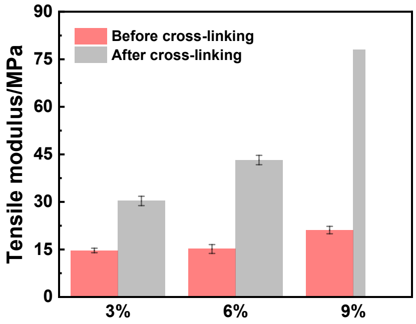

Figure S3. Tensile modulus of PAI/BMI filament before and after crosslinking.

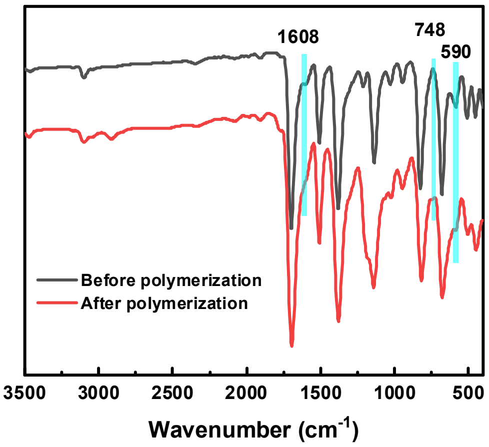

Figure S4. FTIR spectra of BMI powder before and after heating polymerization.

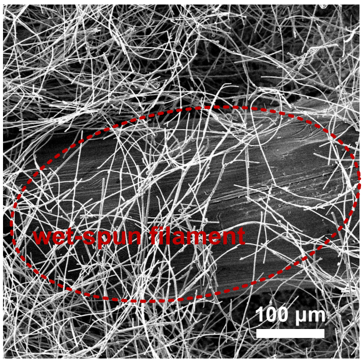

Figure S5. SEM image of dual-scale PAI/PBMI aerogel.

Table S1. The prepared different spinning solutions of wet spinning PAI/BMI filaments

Polymer	Manufacturer and molecular weight	Mass of polymer(g)	Solute weight (DMF)(g)	BMI weight(g)
PAI		1.5	8.5	0
PAI		2.0	8	0
PAI		2.5	7.5	0
PAI	MW=20000, Solvay.	3.0	7	0
PAI		3.5	6.5	0
PAI		3.0	6.7	0.3
PAI		3.0	6.4	0.6
PAI		3.0	6.1	0.9

Supplementary Video

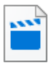

Compression performance dem

The compression recovery property of dual-scale PAI/PBMI fibrous aerogels.

Supplementary Methods

S1. Tensile test of filaments

The tensile properties of the filament were tested by YG005E electronic single yarn strength tester. The filament was held at the two ends of the chuck with 20 mm spacing and drawn at a constant speed of 10 mm min^{-1} until the filament broke. Measure the diameter of the filament using a Vernier scale before testing. Take at least 10 sets of valid data for each sample.

S2. Aerogels compression performance test

The compression performance of aerogel was measured by Keithley 2400 Flexible Material Tester. The compression distance was set according to the height of the sample and the speed was 50 mm min^{-1}. The compression cycle was 1000 times, at least five sets of valid data are retained for each sample

S3. Filtration process

The neutral sodium chloride (NaCl) monodisperse aerosol particles produced from atomizing air pump passed through the test samples with an valid test area of 100 cm^2. The NaCl aerosol particles, featured with a wide range of size from 0.1 to 10 μm, were detected in the upstream and downstream of the tested sample with the quantity of c_1 and c_2, respectively under the ambient temperature of (25 ± 2ºC) and RH of (45 ± 5%). The resulted filtration efficiency could be calculated via $\eta=1-c_1/c_2$. The pressure drop was resulted from the D-value of upstream and downstream measured by flow gauges.
